# Assessing Biomechanical and Clinical Outcomes of an Elbow Orthosis Intervention in Youth Baseball: Preliminary Results

**DOI:** 10.3390/sports12010024

**Published:** 2024-01-09

**Authors:** Gonzalo Mariscal, Carlos Barrios

**Affiliations:** 1 Institute for Research on Musculoskeletal Disorders, Valencia Catholic University, Carrer de Quevedo, 2, 46001 Valencia, Spain; carlos.barrios@ucv.es; 2 Mediterranean Observatory for Clinical and Health Research, Calle San Vicente Mártir, 46017 Valencia, Spain

**Keywords:** orthosis, pitching biomechanics, youth baseball, injury prevention, baseball

## Abstract

Proper orthosis design may help youth baseball players develop safer pitching mechanics to prevent elbow injuries. This study evaluated the impact of a custom elbow orthosis on pitching biomechanics and adverse events. Ten 11–12-year-old players (mean age 11.5 years) from a regional league team were recruited. The inclusion criteria were at least two years of baseball experience. Six players were randomly assigned to the orthosis group, with four in the control group. Anthropometric data and baseline characteristics were recorded. A video analysis assessed elbow flexion angle during pitching at baseline and at 2 months. The frequency of orthosis wear was also tracked. Adverse events during twice-weekly practices were documented. Post-study surveys evaluated orthosis comfort, stability, and safety perceptions. In the orthosis group (n = 6), four participants showed improved elbow flexion angle, and two of the six participants showed almost no change. The overall median difference was 23.5°. In the control group (n = 4), three participants showed improvement, with a median improvement of 5.5°. Twelve adverse events, including pain, were reported by players not wearing orthoses, whereas no events occurred with orthosis use. Individual players in the control group or who did not wear the orthosis correctly experienced multiple episodes of pain from pitching over the study period. This preliminary study indicates a custom elbow orthosis can optimize pitching biomechanics and prevent adverse events in youth baseball players over the course of two months.

## 1. Introduction

Baseball is a common childhood sport in the United States. In 2014, four million children aged 6–12 participated in a baseball team. Shoulder and elbow pain are common complaints among youth pitchers, with 26–51% of players reporting some type of arm pain during the season [1]. In recent decades, many clinical studies have characterized various aspects of Little League elbows. Larson et al. evaluated 120 Minor League pitchers ages 11–12, with an average pitching experience of 2.75 years [2]. They found that 25 of the 120 pitchers (21%) had symptoms related to the dominant elbow, and 35 of the 120 pitchers (29%) showed radiographic changes such as enlargement, fragmentation, separation, or irregularity of the medial epicondyle [2]. Grana et al. evaluated 73 baseball pitchers (average age, 17 years) and found that 58% reported pain while pitching and 56% had radiographic changes [3].

Pitching is one of the fastest movements in sports. Appropriate techniques and biomechanics must be considered when implementing these techniques. The late cocking phase appears to be the critical point in the pitching motion, where the highest levels of torque in the shoulder and elbow can increase the risk of injury [4]. The risk of a youth pitcher suffering a serious injury during a 10-year pitching career is 5%. Limiting the number of innings pitched annually can reduce the risk of injury. Young baseball pitchers are encouraged to play in other positions [5,6]. The number of ulnar collateral ligament reconstructions performed between 2003 and 2014 increased by 343%, and a disproportionate trend in the average annual incidence rate was observed among 15–19-year-old adolescents. Therefore, attempts to prevent such injuries in young pitchers remain paramount [7]. 

Pitching involves various injury patterns that occur because of chronic microtrauma and in vulnerable locations along the skeletally immature elbow. Chronicity, repetitiveness, and valgus overload forces against the medial side of the elbow during adolescence ultimately produce characteristic “Little League elbow” damage. It encompasses a wide variety of pathologies of the medial epicondyle, including apophysitis, avulsion fractures, fragmentation, and growth disturbances (delayed ossification) [8].

Appropriate biomechanics and techniques, as well as training, may help reduce injuries. However, other factors such as repetitiveness, force, anthropometric characteristics, early education, and rehabilitation also play a role in preventing injury mechanisms. An orthosis is a mechanical device that mimics the structure of a joint and is externally applied to assist the movement of the extremities. These instruments are commonly used in rehabilitation programs. A new perspective for addressing elbow pain in young pitchers involves designing an orthosis based on biomechanical analysis of the pitching motion [9]. This orthosis aims to teach proper pitching mechanics, minimize the risk of injuries, and promote optimal techniques while reducing strain on the elbow joint, serving as a re-education tool, and supporting the pitcher during training and games [10,11]. Reviewing the literature, one study observed that the use of an elbow orthosis during repetitive pitching did not reduce range of motion, grip strength, or cause an increased mechanical burden on the throwing arm [10]. Another study also investigated the effects of wearing an elbow orthosis during repetitive pitching in adolescent baseball players and found that while wearing an elbow orthosis did not significantly affect pitching performance, it helped reduce the increase in medial joint space at the elbow, providing relief of stress on the elbow joint during repetitive pitching activities in young players [11].

The objective of this study was to evaluate the effects of a customized elbow orthosis on biomechanical parameters, pain levels, and injury rates in young baseball pitchers. Specifically, we aimed to assess whether the orthosis could (1) enhance the elbow flexion angle during the pitching motion, (2) reduce self-reported pain symptoms, and (3) lower the incidence of elbow injuries compared with participants not using an orthosis. The secondary objective was to evaluate participants tolerance and subjective assessment of orthosis wearing during training and games. 

## 2. Materials and Methods

### 2.1. Study Design

The objective and protocol of this study were approved by the Ethics Committee of the Catholic University of Valencia and adhered to the principles outlined in the Declaration of Helsinki. Written informed consent was obtained from all the participants prior to sample collection. This prospective pilot study was conducted between the years 2019 and 2020.

### 2.2. Eligibility Criteria

The inclusion criteria for this study were as follows: children and adolescents between the ages of 11 and 12 years old; participants who had practiced baseball for a minimum of two years; and participants enrolled in the Los Astros baseball club with parental authorization and coach authorization from the club. The exclusion criteria taken into account for developing this work included: any subjects not meeting the inclusion criteria; failure to sign the informed consent form; those with a musculoskeletal injury preventing adequate exercise performance; and subjects with an illness or condition affecting the throwing motion.

### 2.3. Participants

Initially, there were 15 participants recruited from a youth baseball team in Spain (aged 11 and 12 years) who were playing in a regional baseball league. Of these 15 potential participants, 10 agreed to participate in the study. Given that this study involved 11–12-year-old athletes playing in a regional youth baseball league, where players at this amateur level typically fill various fielding positions and substitute in pitching duties throughout games rather than specializing in a single role, we cannot assume the participants held defined positions like “pitcher” exclusively.

Six participants were randomly assigned to receive the orthosis, and four participants formed the control group. The mean participant age and baseline data are presented in Table 1. At the time of the study, all participants were trained for two days per week (90 min per session). The pace of competitive matches was one per week. However, some participants participated in various sports involving similar throwing mechanisms (softball or golf) or higher-level teams. 

The control group consisted of four participants who met the same inclusion criteria as the experimental group in terms of player age, years of baseball experience, and participation in the Los Astros baseball club. Control subjects did not wear elbow orthoses during pitching. The training and number of throws during the practice sessions were the same in both groups, which was controlled by the team’s coach. This allowed for a proper comparison between conditions with and without orthoses.

### 2.4. Orthosis Design

The elbow orthosis comprises an elastic tubular body made of hypoallergenic elastane and polyamide fabric, dimensioned to fit around the arm, covering the elbow with proximal and distal ends and the anterior, posterior, medial, and lateral sides. It incorporates a means of controlling flexion, including a semirigid foam piece and padding on the anterior side. Adjustable nonrigid means for controlling extension include an inelastic film piece traversing anteriorly from the proximal to distal ends to limit elasticity longitudinally, inelastic straps crossing anteriorly, semirigid spiral rods on the medial and lateral sides imparting a slight bend, and transverse inelastic film pieces on the posterior side, forming an inelastic framework surrounding the central elbow joint posteriorly to limit extension. Additionally, the means for controlling varus–valgus comprise an elastic, adjustable strap transversely on the forearm. Internal medial relief provides targeted pressure on the ulnar collateral ligament during throwing. A tubular body is preferably made from a fabric comprising a hypoallergenic elastane and polyamide blend, wherein the elastic properties of the material are multidirectional/elastic in all directions.

**Figure 1:**
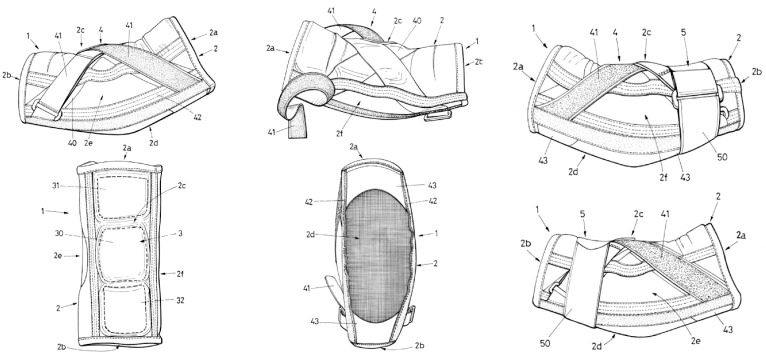
The orthosis used in this study (Figure obtained from the official Spanish Patent and Trademark Register, with permissions. and it is detailed in the Supplementary File S1).

The orthosis was registered by the Spanish Patent and Trademark Office under registration number ES1302006U (Figure 1). A description of the numbers shown in Figure 1 can be found in the Supplementary File S1.

### 2.5. Intervention

Anthropometric data on the elbow were analyzed. The custom-fitted orthosis was manufactured based on the girth measurements of each participant’s elbow. Circumference was recorded using a flexible tape measure wrapped gently around the maximum circumference of the elbow joint, passing over the olecranon and flexed folds. This ensured that the orthosis diameter was appropriately sized for each individual elbow dimension. The surveys were distributed with the assistance of a family member at the initial screening to obtain information on the participants’ history of elbow injuries, weekly frequency of throwing sports practice, medications used, and other illnesses. At the end of the follow-up period, another survey was administered in which participants were asked to subjectively evaluate aspects of using the custom orthosis. Participants were asked to fill out the questions themselves while a parent was present to provide assistance if needed.

Once the participants were assigned an orthosis, video-assisted biomechanical analysis of their throws was performed. A Sony FDR-AX53 4 K (Sony Corporation, Tokio, Japón) camera was used in this study. Video analysis was conducted on the first day of the study and two months after orthosis use. Each participant made three throws, and the average of the three throws was obtained. The camera was placed at chest height. To perform a biomechanical analysis focusing specifically on the elbow joint during throwing, a recording distance of 5 m from each participant was used. At this distance, the angle and movement of the elbow could be captured in clear detail. On the first day, the participants who received orthoses were checked to ensure that the orthoses worked properly. Two months after the start of the study, a second video-assisted assessment was performed. For participants in the orthosis group, the throw was analyzed without an orthosis to determine if the angle had been corrected. During the throwing practice sessions for the orthosis group, the team coach ensured that the players assigned to the orthosis condition wore their orthoses correctly. Before each exercise or throwing session, the coach visually confirmed that each player had an orthosis that was correctly fitted and positioned. Compliance was strictly monitored throughout practice. The coach closely observed the players during the warm-up, throwing mechanics instruction, and all throwing activities to check that the orthosis remained in place. Any player who did not wear the brace for any reason was noted by the coach.

### 2.6. Data Collection and Data Items

**Figure 2:**
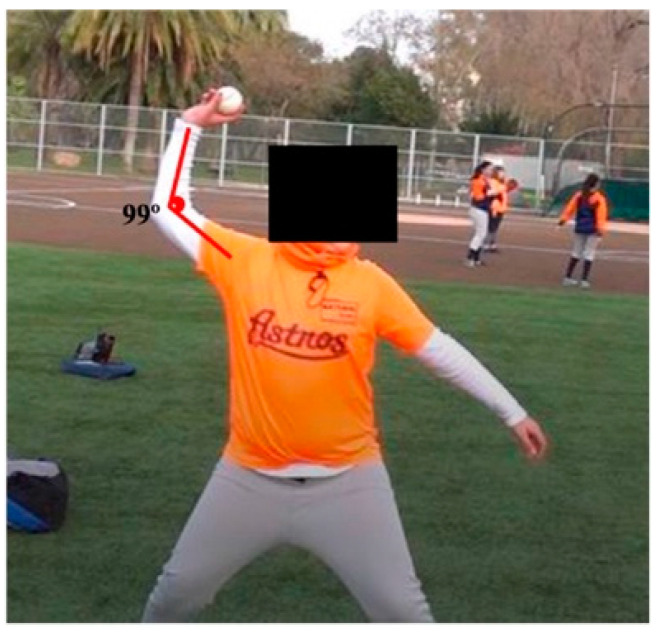
Assessment of the elbow flexion. A 12-year-old male pitching during a training session with an elbow flexion of 99°.

Baseline data: age, weight, days practicing per week, other sports, training time, and pace of competitive matches. The main outcome was elbow flexion (Figure 2). Symptom and injury data were collected during the team’s official training sessions (two days a week—Tuesdays and Fridays). Symptoms and discomfort were recorded for all 10 participants during the follow-up (two months). The elbow was also palpated in participants without symptoms (ulnar collateral ligament and its insertions). 

Finally, a survey was conducted with all participants wearing orthoses to assess comfort (unbearable, uncomfortable, normal, comfortable, and very comfortable), subjective elbow stability (unstable, lack of stability, normal, stable, and very stable), throwing optimization (in all releases, I was throwing as always, I was throwing better), subjective throwing elbow safety (1 = no safety and 5 = high safety), and frequency of orthosis use per week.

### 2.7. Assessment of Results

To evaluate the results of the studies included in this study, an Excel spreadsheet was used in which the most relevant data from each participant were systematically collected. Kinovea-0.9.5 (Kinovea, Bordeaux, France), a validated software, was used to measure elbow flexion angle. After completing the data collection in the spreadsheet, a detailed descriptive review was carried out. Summary tables and figures were created to show and compare the most relevant outcomes. All statistical analyses were performed using IBM SPSS Statistics version 28 (IBM Corp, Armonk, NY, USA). Due to the small sample sizes, non-parametric tests were utilized. A Mann–Whitney U test was conducted to compare flexion angles between the orthoses and control groups. To assess changes in outcomes from before to after the intervention period within each group, the Wilcoxon signed-rank test was utilized. A *p*-value of less than 0.05 was considered statistically significant.

## 3. Results

### 3.1. Baseline Data

A total of 10 participants were included, with 6 initiating orthosis wear (orthosis group) and 4 without (control group). On average, the participants practiced baseball 3–4 times per week. Participants 1–6 wore an orthosis. Participant 5 completed only 20 days with the orthosis before reporting excessive limitation to extension and was moved to the control group at that time point.

### 3.2. Elbow Flexion

In the orthosis group, four of the six participants showed improved angles, and two of the six participants showed almost no change (Table 2). The overall median difference was 23.5°. Eliminating Participant 5, who did not complete the follow-up in the orthosis group, the median correction of the orthosis group was 25.0°. In the control group, three of the four participants showed improvement, with a median improvement of 5.5° (Table 3). Comparisons between the two groups and the assessment of changes in outcomes before and after the intervention period are shown in Table 4.

### 3.3. Clinical Assessment

Twelve adverse events (AEs) were observed. Four players, who initially did not wear an orthosis, reported pain at some point. Participant 5, who was switched to the control group after 20 days of orthosis wear, experienced pain within a week of orthosis removal. Therefore, all five participants (100%) in the control group experienced pain. Of the five participants who remained in the orthosis group for the full two months, one felt pain on the first day of forgetting to wear the orthosis.

In summary, all 12 adverse events of pain or discomfort occurred in participants who did not wear an orthosis, either because they were always in the control group or because the orthosis player forgot the device on the first day. No adverse pain events occurred during the orthosis wear period.

On an individual player basis, player 7 felt pain during four training sessions—three attributed to specific pitches and another due to palpation tenderness. Player 8 had four episodes of pain, one initially and three later in the second month due to improper pitching mechanics (with the last pain lasting 1 week). Player 10 manifested high-intensity elbow pain on pitch and missed 1 week of training. Player 10 also experienced an undefined adverse event without an attributed cause. Upon orthosis removal, player 5 showed focal tenderness on palpation for two consecutive sessions. Player 4 forgot his orthosis on the first assigned day and felt pain during the pitch. 

A visual analysis was conducted to determine whether a relationship existed between patients reporting higher symptomatology and the number of days they practiced per week. Specifically, a significant variation was observed without a clear apparent association, although no statistical analysis was performed. While some individuals with higher symptom scores practiced fewer days, others with similar scores practiced more days. Similarly, patients with lower symptomatology exhibited varied practice frequencies. Due to the lack of statistical evaluation and the visible dispersion of the data, definitive conclusions regarding potential correlations between symptoms and practice load could not be drawn from the descriptive analysis alone.

### 3.4. Survey

**Figure 3:**
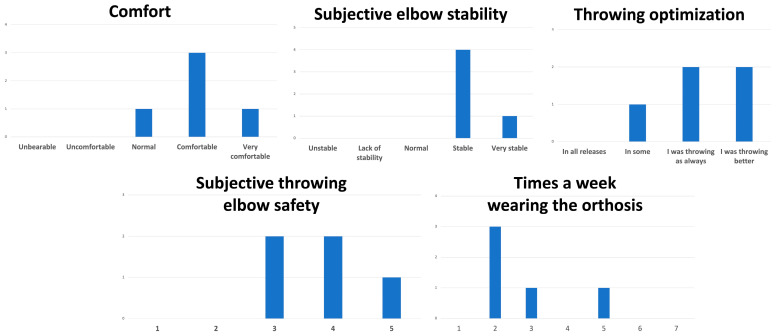
Survey of the participants conducted at the end of the follow-up.

The results of the survey are shown in Figure 3. The survey was conducted with all participants wearing orthoses to assess various factors. Comfort was assessed using a 5-point scale: unbearable, uncomfortable, normal, comfortable, and very comfortable. Subjective elbow stability was also rated on a 5-point scale from unstable to lack of stability from normal to stable to very stable in order to evaluate perceived joint stability. Throwing optimization required participants to indicate whether their throwing was unaffected (I was throwing as always) or improved (I was throwing better) while wearing the orthosis or if it impacted their throwing in all releases. Subjective throwing elbow safety was measured on a 5-point scale, where participants assigned a number from 1 to 5, representing the level of safety felt while throwing, with 1 indicating no safety and 5 representing high safety. Finally, to capture utilization, participants reported the average frequency of orthosis use per week for their sporting activities. This comprehensive survey systematically evaluated comfort, elbow stability, effects on throwing performance, feelings of safety, and orthosis utilization from the participants’ perspective through explicit rating scales and descriptions for each factor assessed.

## 4. Discussion

The present study substantiates the requirement for additional interventions during pitching education for youth baseball. Orthotics could improve pitching angle and reduce adverse events. The current study found that angle correction was more pronounced in participants who used orthoses, although there were no significant differences. The literature on baseball pitching recommends angles less than 90° for safer and more efficient throwing [12]. In this study, all players in the orthosis group were initially pitched at angles of less than 90° before using the orthosis [11]. When the elbow flexion angle is less than 90°, there is a risk of injury, necessitating additional corrective mechanisms during training. After two months with the orthosis, four participants showed considerable correction, with three of them moving out of the high-risk zone of flexions greater than 90°. Pitching mechanics guidelines recommending correct techniques are well established [13], yet not all young pitchers naturally achieve ideal angles, indicating that supplemental intervention may be warranted [13]. The use of the orthosis appeared to influence pitching mechanics by helping move the angles closer to the recommended safe levels. This study provided initial evidence that orthosis use may help pitching form.

The two participants who did not show correction were those who initially pitched at the extremely closed angles of 36° and 39°. This could indicate that, in cases of pitches with such acute angles, re-evaluation of pitching mechanics may be necessary for these players. Combining orthoses with pitching education may lead to corrections. It is also possible that there was insufficient time for the correction to occur. These results agree with evidence demonstrating a relationship between abnormal pitching biomechanics and greater injury risk, particularly in youth with developing musculoskeletal systems [14]. In cases of extreme pitching deviations, alternative corrective options, such as modifying pitching styles, may prove more suitable than orthotics alone for correcting faulty movement patterns [15]. Longer intervention periods combining orthosis wear, real-time feedback devices, and instructions from trained coaches also present a promising multimodal approach worthy of future investigation [16].

The findings related to player 5 achieving angle modification after a relatively brief 20-day trial of orthosis wear are consistent with reports that demonstrated measurable biomechanical changes within comparable treatment periods. These preliminary findings suggest that orthotics have the potential to influence pitching mechanics over a short period of time. Long-term orthosis therapy may confer more lasting protection against injury while preserving functional performance.

The observed differences in subjective symptom reports and physical examination findings between the control and orthosis groups enhance our understanding of overuse injury mechanisms in youth pitchers. Specifically, our hypothesized contribution of accumulated fatigue (the loss of muscle force-generating capacity due to repetitive physical exertion) from repetitive arm motions aligns with substantial literature identifying heavy workload volume without adequate rest as a modifiable risk factor for pain and soft tissue damage in the shoulder and elbow. Orthoses appear to be capable of mitigating this dose-dependent injury progression by reducing joint and muscular stresses with each pitch as the pitching exposure increases. Strategies balancing pitching participation with rest, guided by modern scientific pitching guidelines, are critical.

Considering the number of pitches or repetitiveness of elbow flexion movements during pitching, player 7 pitched at a marginally acceptable (yet risky) angle and experienced more pain throughout the second month. However, his pitching frequency was seven days per week. Therefore, orthosis use could optimize pitch count, raising the threshold for the number of pitches required to induce pain or injury.

Orthoses counteract excessive valgus moments and abnormal elbow extension torque, which are otherwise associated with fatigue and overuse [17]. Given that microtrauma inevitably accumulates with the high training volumes common in youth sports, bracing appears to be protective for this at-risk population by dampening stress with each throw.

At the end of the follow-up survey, the orthosis was not uncomfortable for any participant. Even though the orthosis design did not prioritize comfort, player satisfaction was achieved. The orthosis fulfilled one of its key intended functions by subjectively enhancing perceptions of stability and safety during pitching motions. Players felt secure during pitching. The participants felt that the orthosis was effective.

The hypothesis that perceptions of enhanced stability and safety may enable improved performance is supported by evidence that optimized biomechanics underlie skill progression [18,19,20]. Orthoses may guide proper arm alignment to facilitate motor learning while sparing tissues from excessive force pitching techniques. Simultaneously, bracing may relieve distracting elbow soreness, which interferes with competitive focus.

The survey question regarding “elbow safety” served to corroborate whether players’ self-assessed stability accurately reflected their pitching risks. If stability is perceived as good, elbow safety during pitching motions should be followed. Therefore, responses to this confidence query confirmed the stability and safety perceptions of players associated with bracing.

Given an average of 3–4 training sessions or matches per week for these athletes, wearing the orthosis for 2 days did not induce discomfort and has the potential to prevent both acute and overuse injuries, with all implications for development considered. However, two players utilized bracing for 3–5 days weekly.

A comparison of the results of this study with those of other studies has shown some similarities and differences. Two studies reported that orthosis use did not significantly affect pitching performance [9,10]. However, one of these studies observed that orthosis provided relief from stress on the elbow joint during repetitive pitching activities. In contrast, our exploratory findings suggested that orthoses may have positive benefits for symptom reduction and pitching performance, although our conclusions acknowledge the speculative nature due to limitations. Notably, our study examined a younger population of young baseball players, whereas the reference studies involved older adolescent athletes.

### 4.1. Limitations of the Study

The main limitation of this pilot study was its small sample size, which reduced the consistency and generalizability of the conclusions. Additionally, as the study was conducted over a short period of only two months, a longer follow-up is needed to more rigorously assess the durability of orthosis effectiveness both in the short and long term. The study period could not be extended due to limitations imposed by the youth sports team to minimize disruption to players’ training routines. The heterogeneous and multifactorial nature of pitching biomechanics and injury risk presents interpretive challenges, as there are numerous potentially confounding variables that cannot be fully controlled. Although the control group was matched to key variables, such as age, experience, and team, the study design did not fully account for individual differences that could influence the results. In particular, factors such as physical maturity, injury history, and differences in motivation/feedback between practice settings were not measured, which could have introduced unbalanced variability between the groups. Overall, although important sources of bias were minimized, the risk of some residual confounding cannot be ruled out, given that not all relevant individual characteristics were accurately measured and controlled for between the experimental and control participants. Another limitation of the study was that the participants were young athletes engaged in recreational sporting activities, in part for enjoyment, and not in a strictly controlled laboratory setting. More stringent criteria for fitting and continued monitoring of orthosis use are needed in future research to fully control the intervention. The less stringent bracing protocols used in the current study reflect real-world conditions in youth sports but reduce the ability to definitively attribute outcomes specifically to the effects of the orthosis. Objective measurement biases may also have influenced some subjective outcome assessments such as adverse event reporting and survey responses. Another limitation of the current study is that the potential impact of the orthosis on pitching performance was assessed through subjective coach/player ratings rather than objective performance metrics, such as velocity, accuracy, or scoring. While qualitative feedback provides insight, definitive conclusions cannot be drawn without more rigorous outcome measures. Larger sample sizes employing quantitative performance data are required to validate any effects on the actual on-field ability. Furthermore, the development of biomechanics in adolescent athletes introduces complexity compared to that in adult athletes. Fatigue was conceptualized as the loss of muscle force-generating capacity due to repetitive physical exertion. This definition captures the temporary decrease in the muscle force-generating capacity that arises from repeated strenuous muscle contractions over time. Although no formal assessment was used, given the preliminary nature of our study, future work may benefit from the use of validated metrics to objectively quantify changes in dynamic and/or isometric strength to complement self-reported perceptions of muscle fatigue or exhaustion. These limitations constrain causal inferences and highlight the need for larger studies with standardized objective outcome measures collected over extended longitudinal follow-ups to validate any effects and account for potential confounding influences in this population.

### 4.2. Conclusions and Future Recommendations

In conclusion, based on the findings of this study, orthoses show promise as an effective intervention to reduce injury risk and improve pitching performance in youth baseball. The orthosis demonstrated positive effects in correcting pitching angles and enhancing players’ confidence and performance. Additionally, orthosis was well tolerated and accepted by most players in this study. However, it is important to note that the conclusions drawn regarding the long-term effects and injury prevention are speculative because of the limited evidence from this study. Therefore, further research is warranted to explore the long-term effects of orthosis on pitching performance and injury prevention. It would be beneficial to investigate the effectiveness of orthoses in conjunction with specific training programs to optimize pitching techniques. Furthermore, it is recommended to study the impact of orthoses on players with closed pitching angles, as our study suggests that these players may require a different approach or additional time for correction.

## Figures and Tables

**Table 1:** Baseline characteristics of the 10 included participants.

Participant	Age	Weight (kg)	Body Height (cm)	BMI	Days Practicing per Week
Participant 1	12	60	160	21.6	5
Participant 2	11	45	150	20.0	2
Participant 3	12	30	145	23.5	2
Participant 4	12	50	145	18.5	4
Participant 5	11	36	138	21.8	4
Participant 6	12	48	148	24.9	4
Participant 7	11	53	145	37.0	7
Participant 8	12	89	155	24.5	3
Participant 9	12	55	158	22.0	4
Participant 10	11	35	139	18.1	2

**Table 2:** Elbow flexion values in the orthosis group.

Orthosis Group	Before	2 Months	Difference
Participant 1	76°	114°	38°
Participant 2	36°	33°	−3°
Participant 3	76°	104°	28°
Participant 4	66°	91°	25°
Participant 5	56°	78°	22°
Participant 6	39°	38°	−1°

**Table 3:** Elbow flexion values in the control group.

Control Group	Before	2 Months	Difference
Participant 7	72°	78°	6°
Participant 8	72°	81°	9°
Participant 9	90°	80°	−10°
Participant 10	90°	95°	5°

**Table 4:** Comparison between the two groups.

Group	Before	2 Months	Difference	*p*-Value
Orthosis group	59°	76°	17°	0.059
Control group	81°	84°	3°	0.886
*p*-value	0.085	0.915	0.201	

## Data Availability

For those interested, the data from this study can be obtained by contacting the corresponding author.

## Supplementary Materials

The following supporting information can be downloaded at: https://www.mdpi.com/article/10.3390/sports12010024/s1, Figure S1: Orthosis design.

## References

[1] Albright J.A., Jokl P., Shaw R., Albright J.P. (1978). Clinical study of baseball pitchers: Correlation of injury to the throwing arm with method of delivery. Am. J. Sports Med..

[2] Larson R.L., Singer K.M., Bergstrom R., Thomas S. (1976). Little League survey: The Eugene study. Am. J. Sports Med..

[3] Grana W.A., Rashkin A. (1980). Pitcher’s elbow in adolescents. Am. J. Sports Med..

[4] Anz A.W., Bushnell B.D., Griffin L.P., Noonan T.J., Torry M.R., Hawkins R.J. (2010). Correlation of torque and elbow injury in professional baseball pitchers. Am. J. Sports Med..

[5] Fleisig G.S., Andrews J.R., Cutter G.R., Weber A., Loftice J., McMichael C., Hassell N., Lyman S. (2011). Risk of serious injury for young baseball pitchers: A 10-year prospective study. Am. J. Sports Med..

[6] Thompson S.F., Guess T.M., Plackis A.C., Sherman S.L., Gray A.D. (2018). Youth Baseball Pitching Mechanics: A Systematic Review. Sports Health.

[7] Mahure S.A., Mollon B., Shamah S.D., Kwon Y.W., Rokito A.S. (2016). Disproportionate trends in ulnar collateral ligament reconstruction: Projections through 2025 and a literature review. J. Shoulder Elb. Surg..

[8] Brogdon B.S., Crow M.D. (1960). Little Leaguer’s elbow. Am. J. Roentgenol..

[9] Wicke J., Keeley D.W., Oliver G.D. (2013). Comparison of pitching kinematics between youth and adult baseball pitchers: A meta-analytic approach. Sports Biomech..

[10] Hattori H., Akasaka K., Otsudo T., Hall T., Amemiya K., Mori Y. (2019). Use of an Elbow Brace During Repetitive Pitching Does Not Cause an Increased Mechanical Burden on the Throwing Arm. PM R.

[11] Hattori H., Akasaka K., Otsudo T., Takei K., Yamamoto M. (2017). The Effects of Elbow Bracing on Medial Elbow Joint Space Gapping Associated with Repetitive Throwing in High School Baseball Players. Orthop. J. Sports Med..

[12] Lyman S., Fleisig G.S., Andrews J.R., Osinski E.D. (2002). Effect of pitch type, pitch count, and pitching mechanics on risk of elbow and shoulder pain in youth baseball pitchers. Am. J. Sports Med..

[13] Parks E.D., Ray T.R. (2009). Prevention of overuse injuries in young baseball pitchers. Sports Health.

[14] Shanley E., Rauh M.J., Michener L.A., Ellenbecker T.S., Garrison J.C., Thigpen C.A. (2011). Shoulder range of motion measures as risk factors for shoulder and elbow injuries in high school softball and baseball players. Am. J. Sport. Med..

[15] Wilk K.E., Reinold M.M., Andrews J.R. (2004). Rehabilitation of the thrower’s elbow. Clin. Sports Med..

[16] Lizzio V.A., Cross A.G., Guo E.W., Makhni E.C. (2020). Using Wearable Technology to Evaluate the Kinetics and Kinematics of the Overhead Throwing Motion in Baseball Players. Arthrosc. Tech..

[17] Sabick M.B., Torry M.R., Lawton R.L., Hawkins R.J. (2004). Valgus torque in youth baseball pitchers: A biomechanical study. J. Shoulder Elb. Surg..

[18] Fleisig G.S., Barrentine S.W., Zheng N., Escamilla R.F., Andrews J.R. (1999). Kinematic and kinetic comparison of baseball pitching among various levels of development. J. Biomech..

[19] Keeley D.W., Hackett T., Keirns M., Sabick M.B., Torry M.R. (2008). A biomechanical analysis of youth pitching mechanics. J. Pediatr. Orthop..

[20] Dun S., Loftice J., Fleisig G.S., Kingsley D., Andrews J.R. (2008). A biomechanical comparison of youth baseball pitches: Is the curveball potentially harmful?. Am. Sports Med..

