# Supplementary material for: Assessing Biomechanical and Clinical Outcomes of an Elbow Orthosis Intervention in Youth Baseball: Preliminary Results
Source: Sports (Basel). 2024 Jan 9;12(1):24. doi: 10.3390/sports12010024 (PMC10819216; doi:10.3390/sports12010024)
Supplement: Supplementary file 1 [file sports-12-00024-s001.zip › sports-2753355-supplementary.pdf]

Supplementary File S1. Orthosis design.

In view of the aforementioned figures, a possible example of an elbow orthosis, preferably for the prevention of injuries in athletes, is described below.
Thus, as can be seen in the figures, the elbow orthosis (1) of the invention is essentially composed of a tubular body (2) preferably made of elastic fabric, which is sized to adapt to the user's arm, covering the elbow, with a proximal end (2a) corresponding to the upper edge that fits on the arm, a distal end (2b) corresponding to the lower edge that fits the forearm, an anterior side (2c) corresponding to the front of the elbow, a posterior side (2d) corresponding to the back of the elbow, a medial side (2e) corresponding to the inside of the elbow, and a lateral side (2f) corresponding to the outside of the elbow.
From this known configuration, tubular body (2) comprises, incorporated therein, at least:
- means of control or limitation of flexion (3), preferably comprising at least a central piece of semi-rigid foam (30) and padding (31, 32) such that, as shown in Figure 2, they cover the entire area of the front side (2c) of the tubular body (2).
- and means of control, or limitation, of extension (4) in an adjustable manner, by means of non-rigid materials that, at least preferably, comprise a piece of inelastic film in the shape of a cross (40) (figure 3), which is incorporated on the anterior side (2c) of the tubular body, runs along the orthosis from the area of the proximal end (2a) to the area of the distal end (2b), keeping the tubular body (2) in slight flexion and limiting the elasticity of the fabric longitudinally.

The central piece of the semi-rigid foam (30) is approximately 10 mm thick and is fixed inside an inner pocket. Likewise, the padding (31, 32), which, together with the central piece (30), covers the entire area of the front side (2c) of the tubular body (2), is formed by an upper piece (31) of foam and a lower piece (32) of foam located in the respective pockets of the tubular body (2).
Preferably, the extension control means that (4) further comprises two inelastic straps (41) (Figures 1, 3, and 5) that cross the front side (2c) of the tubular body (2) from medial (2e) to lateral (2f) in the longitudinal direction over the blade-shaped film piece (40), which close on themselves, being fixed in an adjustable manner by means of a hook-and-loop fastener.
Extension control means that (4) also comprises two semi-rigid spiral rods (42) (Figures 1 and 5) located inside their respective pockets, one on the medial side (2e) and the other on the lateral side (2f), which also have a slight curvature that maintains the tubular body (2) of orthosis (1) in slight flexion.
The extension control refers to (4), which allows the extension to be adjusted, and also comprises two transversal pieces of inelastic film (43) (Figure 4), fixed to the tubular body (2) on the rear side (2d) near the proximal (2a) and distal (2b) ends, which cross transversely through the tubular body (2) from the medial side (2e) to the lateral side (2f). Both transverse pieces of inelastic film (43) have a curved edge on the side where they face each other, such that they define an oval-shaped central area of the tubular body (2) on its rear side (2d), which is not covered.
In any case, the set of elements comprising the joint extension control means that (4) defines a non-rigid inelastic frame around the central area of the rear side (2d) of the tubular body (2), that is, in correspondence with the elbow joint.
Likewise, and preferably, orthosis (1) also comprises means of control or limitation of the varus-valgus (5) of the elbow, consisting of at least an adjustable elastic strap (50) (Figures 5 and 6) that fits transversely over the tubular body (2) in the part of the forearm close to the distal end (2b), to control the deviations in valgus of the joint.
In addition, the orthosis includes a small relief or support sewn on the inside, specifically on the inside of the medial side (2e), which exerts pressure on a point inside the elbow, specifically on the ulnar collateral ligament. This pressure point relieves pressure on the ligament when stretching the arm during a ball throw; for example, when playing a sport such as baseball, it helps to reduce stress and fatigue on the ligament without reducing its functionality.
The material from which the tubular body (2) is made is preferably an elastic fabric of hypoallergenic elastane and polyamide, in which the elasticity of the material is multidirectional.

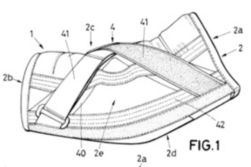

FIG.1

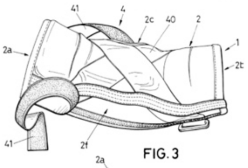

FIG.3

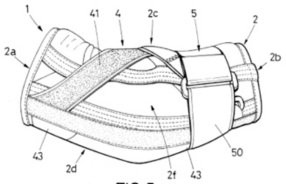

FIG.5

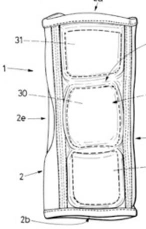

FIG.2

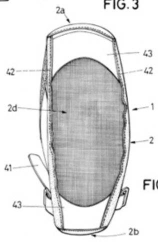

FIG.4

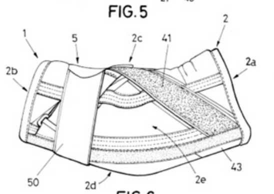

FIG.6
